# Development of a collapsed cone convolution/superposition dose calculation algorithm with a mass density-specific water kernel for magnetic resonance-guided radiotherapy

**DOI:** 10.1093/jrr/rrad011

**Published:** 2023-03-21

**Authors:** Kengo Ito, Yojiro Ishikawa, Satoshi Teramura, Noriyuki Kadoya, Yoshiyuki Katsuta, Shohei Tanaka, Ken Takeda, Keiichi Jingu, Takayuki Yamada

**Affiliations:** Division of Radiology, Tohoku Medical and Pharmaceutical University, Sendai, Miyagi 983-8536, Japan; Department of Radiation Oncology, Tohoku University Graduate School of Medicine, Sendai, Miyagi 980-8574, Japan; Division of Radiology, Tohoku Medical and Pharmaceutical University, Sendai, Miyagi 983-8536, Japan; Division of Radiology, Tohoku Medical and Pharmaceutical University, Sendai, Miyagi 983-8536, Japan; Department of Radiation Oncology, Tohoku University Graduate School of Medicine, Sendai, Miyagi 980-8574, Japan; Department of Radiation Oncology, Tohoku University Graduate School of Medicine, Sendai, Miyagi 980-8574, Japan; Department of Radiation Oncology, Tohoku University Graduate School of Medicine, Sendai, Miyagi 980-8574, Japan; Department of Radiological Technology, School of Health Sciences, Faculty of Medicine, Tohoku University, Sendai, Miyagi 980-8575, Japan; Department of Radiation Oncology, Tohoku University Graduate School of Medicine, Sendai, Miyagi 980-8574, Japan; Division of Radiology, Tohoku Medical and Pharmaceutical University, Sendai, Miyagi 983-8536, Japan

**Keywords:** magnetic field, magnetic resonance-guided radiotherapy, fast dose calculation, collapsed cone convolution/superposition, energy deposition kernel

## Abstract

This study aimed to develop and validate a collapsed cone convolution for magnetic resonance-guided radiotherapy (CCC_MR_). The 3D energy deposition kernels (EDKs) were generated in water in a 1.5-T transverse magnetic field. The CCC_MR_ corrects the inhomogeneity in simulation geometry by referring to the EDKs according to the mass density between the interaction and energy deposition points in addition to density scaling. Dose distributions in a water phantom and in slab phantoms with inserted inhomogeneities were calculated using the Monte Carlo (MC) and CCC_MR_. The percentage depth dose (PDD) and off-axis ratio (OAR) were compared, and the gamma passing rate (3%/2 mm) was evaluated. The CCC_MR_ simulated asymmetric dose distributions in the simulation phantoms, especially the water phantom, and all PDD and OAR profiles were in good agreement with the findings of the MC. The gamma passing rates were >99% for each field size and for the entire region. In the inhomogeneity phantoms, although the CCC_MR_ underestimated dose in the low mass density regions, it could reconstruct dose changes at mass density boundaries. The gamma passing rate for the entire region was >95% for the field size of 2 × 2 cm^2^, but it was 68.9–86.7% for the field sizes of ≥5 × 5 cm^2^. Conclusively, in water, the CCC_MR_ can obtain dose distributions comparable to those with the MC. Although the dose differences between them were mainly in inhomogeneity regions, the possibility of the effective use of the CCC_MR_ in small field sizes was demonstrated.

## INTRODUCTION

Magnetic resonance (MR)-guided radiotherapy (MRgRT) was developed to improve treatment accuracy, and MR images are used to reduce setup uncertainty without additional imaging dose. Because MR images provide high soft tissue contrast, MRgRT can directly localize the tumor [1]. Furthermore, MR images have been used to monitor treatment response and biological or functional images [2, 3]. MRgRT systems allow online adaptive radiation therapy (online-ART) for treatment adjustment according to the conditions of tumor volume or the organs at risk based on pretreatment assessment [4, 5].

Treatment planning systems for MRgRT use Monte Carlo (MC) simulation to obtain the dose distributions in magnetic fields because MC simulation can accurately simulate particle transport in a magnetic field [6, 7]. One of the drawbacks of MC simulation is that it requires a long calculation time to obtain sufficient statistical significance; however, it is becoming clinically acceptable. Although MC simulation uses variance reduction techniques and a graphical processing unit (GPU) to reduce the calculation time, a certain calculation time is required to obtain the dose distribution if the optimization step of intensity-modulated radiation therapy (IMRT) is included [8]. Some researchers revealed the planning time for online-ART in MRgRT. Raaymakers *et al*. reported that Unity 1.5 T (Elekta AB, Sweden) requires ~ 5 min for their concept, and Acharya *et al*. showed that the median ART time for MRIdian (Viewray, Inc, Cleveland, OH) was 26 min, including the recontouring, reoptimization and QA steps [4, 9]. Moreover, Muinck Keizer *et al*. reported that patients with prostate diseases had an average on-couch time of 40 min during a single fraction, and targets, such as the prostate and seminal vesicle, had intrafraction motion of a few millimeters 10 min after the first cine-MR dynamic image acquired in that fraction [10]. Circumstances should be created to allow the immediate start of treatment beam irradiation after imaging or creating a treatment plan on online-ART to reduce variations in the patient’s body.

Collapsed cone convolution (CCC)/superposition dose calculation algorithms that use the precalculated energy deposition kernels (EDKs) by MC simulation can obtain dose distributions faster than MC methods because of the amount of computation. Furthermore, CCC algorithms can obtain the dose distributions within a few seconds with the use of GPUs [11, 12]. If CCC algorithms could be applied to MRgRT, we would be able to reduce the patient’s waiting time associated with dose calculation as well as improve the intra-fractional errors. The major difference from the conventional situation without a magnetic field is that it is necessary to reproduce the dose variations caused by the Lorentz force in a magnetic field. In particular, the direction of the field is orthogonal to the treatment beam, and non-negligible dose changes are caused by the electron return effect (ERE) at mass density change regions, such as the body surface and hollow organs [13]. General CCC algorithms assume that the human body is constructed with water and calculated the dose to water. The EDK obtained in water is discretized into a cone shape with the origin at the interaction point to reduce the amount of calculation and achieve high speed. Moreover, inhomogeneity correction is performed by scaling the length based on the mass density on the path of each cone [14]. EDKs were acquired not in water but in arbitrary body tissues and materials (material-specific kernel) and used for CCC algorithms to improve the dose calculation accuracy in not only in the MV domain but also in the kV domain [15, 16]. The use of EDKs obtained from arbitrary materials improves dose calculation accuracy because the reproducibility of particle transport in arbitrary materials can be improved.

In this study, we generated EDKs obtained from water with varying mass densities (mass-specific EDK [EDK_ms_]) to consider that the trajectory of particles in a magnetic field varies with the mass density of water. We attempted to obtain the dose distributions in a transverse magnetic field by using the CCC algorithm with EDK_ms_ and validated the accuracy of the CCC algorithm with EDK_ms_ compared with MC dose distributions.

## MATERIALS AND METHODS

### Generation of the EDK_ms_ in a magnetic field

**Fig. 1:**
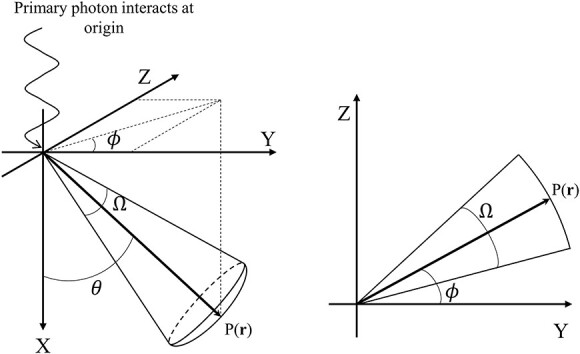
Schematic of the coordinate system for the generation of EDKs in a magnetic field. Monoenergetic primary photons interact at the origin, which is the center of the water sphere. P(**r**) indicates the position of the particle, and *θ* and *ϕ* are the angles between the vector **r** and the positive X or Y axis. Ω is the angular boundary as the steradian.

MC code Electron Gamma Shower version 5 (EGS5) was used to obtain EDKs in a magnetic field, and the accuracy of this process in a magnetic field was evaluated according to the method by Ito *et al*. [17]. We used a homogeneous sphere of water with a radius of 60 g·cm^−2^ to obtain EDKs. The radial boundaries of voxels were defined at the intersection of 65 radial shells. The smallest radial spacing was 0.01 g·cm^−2^, which occurs immediately surrounding the origin, and the largest radial spacing was 5.0 g·cm^−2^, which occurs near the edge of the phantom. As a high-dose gradient would be observed near the site of the incident photon, high-resolution scoring boundaries were defined. Conversely, as a roughly equal amount of energy deposition would be observed on the distal side in the phantom, low-resolution scoring boundaries were defined. Generally, the CCC method uses the kernels obtained by discretizing the 3D EDKs into 2D polar coordinates, since a symmetrical point spread dose distribution is produced in the homogeneous media without a magnetic field. However, the EDKs in a magnetic field are expected to be distorted and affected by the Lorentz force depending on the relationship between the direction of the incident primary photon and the magnetic field. In addition, it is straightforward to assume that the reconstruction of the distorted dose distribution in a magnetic field from 2D general EDKs is extremely challenging. Hence, to be able to consider the distorted EDKs in a magnetic field, the angular boundaries (Ω) were defined by dividing 4π steradian (sr) into 0.0086 sr (=6°) to construct 3D distorted EDKs. Figure 1 shows a schematic of the simulation geometry.

Water has a mass density of 1 g·cm^−3^ under standard conditions. In this study, to consider that the trajectory of the charged particle is dependent on the mass density of the medium in the pathway, we obtained EDKs generated in the water sphere, which had a mass density of 0.25 g·cm^−3^ at intervals between 0.25 and 2.0 g·cm^−3^. Hence, eight EDKs were obtained per monoenergetic primary photon. The generated EDKs were specific to the mass density of water; hence, we defined them as EDKs_ms_. The radii of the water spheres were scaled based on the mass density of each simulation so that the product of the radius and mass density was the same for all simulations (e.g. *r*_1.0_ × *ρ*_1.0_ = *r*_0.25_ × *ρ*_0.25_; subscripts mean the mass density [g·cm^−3^]). The monoenergetic EDKs_ms_ from 1.0 to 7.0 MeV were obtained at 1.0 MeV intervals. The strength of the magnetic field was set to 1.5 T, and the direction of the field was perpendicular to the incident primary photon direction. The relationship between the direction of the primary photon and the field assumed that the treatment beam and field direction are orthogonal in MRgRT.

The history number was set to 1 × 10^8^ to generate each EDK_ms_. The EDK_ms_ and statistical uncertainty of the MC simulation were determined for each calculated quantity by dividing the calculations into 10 batches and computing the mean and root mean square deviation from the mean from these 10 values. Furthermore, to characterize the variation in the EDK_ms_ depending on the mass density, the effective depth ($\overline{z}$), effective radial distance ($\overline{r}$) and effective lateral distance ($\overline{y}$) were obtained for each EDK_ms_. These parameters explained the average traveling distance of the charged particle from the interaction point.

The effective radial distance $\overline{r}$ is described as follows:


(1) \begin{equation*} \overline{r}=\sum_i\sum_j\frac{{\hat{r}}_{\mathrm{prim}}\left(i,j\right){\varepsilon}_{\mathrm{prim}}\left(i,j\right)}{F_{\mathrm{prim}}} \end{equation*}

where ${\varepsilon}_{\mathrm{prim}}\left(i,j\right)$ is the energy deposition of the primary charged particle of the *i*, *j*th voxel. Furthermore, the effective radius of each voxel ${\hat{r}}_{\mathrm{prim}}\left(i,j\right)$ by the primary charged particle is described as follows:


(2) \begin{equation*} {\hat{r}}_{\mathrm{prim}}\left(i,j\right)=\sum_n{r}_n{\left[{\varepsilon}_{\mathrm{prim}}\left(i,j\right)\right]}_n{\left(\sum_n{\left[{\varepsilon}_{\mathrm{prim}}\left(i,j\right)\right]}_n\right)}^{-1} \end{equation*}

where *r*_n_ is the distance from the origin of the sphere to the center of the *i*, *j*th voxel, and *n* is the number of MC iterations. The effective depth $\overline{z}$ is described as follows:


(3) \begin{equation*} \overline{z}=\sum_i\sum_j\frac{{\hat{z}}_{\mathrm{prim}}\left(i,j\right){\varepsilon}_{\mathrm{prim}}\left(i,j\right)}{F_{\mathrm{prim}}} \end{equation*}

where ${\hat{z}}_{\mathrm{prim}}\left(i,j\right)$ is the effective depth of each *i*, *j*th voxel by the primary charged particle, which is described as follows:


(4) \begin{equation*} {\hat{z}}_{\mathrm{prim}}\left(i,j\right)={\hat{r}}_{\mathrm{prim}}\left(i,j\right)\cos \left[{\hat{\theta}}_{\mathrm{prim}}\left(i,j\right)\right] \end{equation*}

(5) \begin{equation*} \cos \left[{\hat{\theta}}_{\mathrm{prim}}\left(i,j\right)\right]=\sum_n\cos \left({\theta}_n\right){\left[{\varepsilon}_{\mathrm{prim}}\left(i,j\right)\right]}_n{\left(\sum_n{\left[{\varepsilon}_{\mathrm{prim}}\left(i,j\right)\right]}_n\right)}^{-1} \end{equation*}

where $\cos \big[{\hat{\theta}}_{\mathrm{prim}}\left(i,j\right)\big]$ is the cosine value of the effective angle ${\hat{\theta}}_{\mathrm{prim}}\left(i,j\right)$ of each *i*, *j*th voxel by the primary charged particle, which is the angle between the direction of the incident primary photon and the middle of the voxel of the particle position. The effective lateral distance $\overline{y}$ is obtained from $\overline{r}$ and $\overline{z}$, and is described as follows:


(6) \begin{equation*} \overline{y}=\sqrt{{\overline{r}}^2-{\overline{z}}^2} \end{equation*}

The ratio of $\overline{z}/\overline{y}$, which indicates whether the charged particles deposit their energy in the forward or lateral direction, and the shape of the EDK_ms_ were obtained [15, 16, 18]. The cutoff energies for photons and electrons were set to 0.01 and 0.521 MeV, respectively. The EFRACH/EFRACL variables, which specify the fractional energy losses over multiple scattering steps at the highest or lowest problem energies, respectively, were set at 0.02. The maximum user step length (‘mxustep’) of the charged particles was set to 0.001 cm.

### C‌CC/superposition dose calculation algorithm and inhomogeneity correction

The original convolution/superposition dose calculation (C/S) algorithm was expressed using the following equation:


(7) \begin{equation*} D\left(\mathbf{r}\right)=\int \int \int \int{T}_E\left(\mathbf{s}\right){h}_{\rho_0}\left(E,\mathbf{r}-\mathbf{s}\right){\mathrm{d}}^3s\mathrm{d}E \end{equation*}

where **r** is a point at which a dose is calculated, and **s** is the source point of the energy imparted to **r**. *T_E_* indicates the total energy released by primary photon interactions per unit mass (TERMA) and is expressed as follows:


(8) \begin{equation*} {T}_E\left(\mathbf{r}\right)=\int \frac{\mu }{\rho}\left(E,\mathbf{r}\right)E{\Phi}_E\left(\mathbf{r}\right)\mathrm{d}E \end{equation*}

where $\mu /\rho \left(E,\mathbf{r}\right)$ is the mass attenuation coefficient of the primary photons of energy *E* at point **r,** and ${\Phi}_E\left(\mathbf{r}\right)$ is the primary photon fluence differential in energy at **r**. Because the photon attenuation is not affected by a magnetic field, the TERMA distribution can be obtained using the general method. We assumed that the TERMA is constant in one voxel because the TERMA usually varies slowly [19]. In addition, ${h}_{\rho_0}$ indicates the point spread EDK in a homogeneous medium of density ${\rho}_0$. The vector (**r**–**s**) is rescaled according to the mass density on each path length to consider inhomogeneous correction [14]. The CCC algorithm is an approximation of the C/S algorithm and uses the EDK that is collapsed to a discrete number of directions. The complexity of the CCC algorithm is *O*(*N*^4^*M*) for arbitrary EDK, where *M* is the number of collapsed cone directions with inhomogeneity correction, whereas that of the C/S algorithm is *O*(*N*^7^), which is significantly more than that of the CCC algorithm [20]. As the CCC algorithm requires less calculation, it can quickly obtain the dose distributions.

In addition to the radiological path length from **s** to **r** (${p}_{\mathbf{s},\mathbf{r}}$), the CCC algorithm developed by us for MRgRT (collapsed cone convolution for magnetic resonance-guided radiotherapy [CCC_MR_] algorithm) obtained the average mass density from **s** to **r** (${\overline{\rho}}_{\mathbf{s},\mathbf{r}}$) for the inhomogeneity correction. The energy deposition from **s** to **r** was obtained from the EDK_ms_(${\overline{\rho}}_{\mathbf{s},\mathbf{r}},{p}_{\mathbf{s},\mathbf{r}}$) generated in a water sphere with mass density ${\overline{\rho}}_{\mathbf{s},\mathbf{r}}$. In case EDK_ms_(${\overline{\rho}}_{\mathbf{s},\mathbf{r}}$) was not generated, the CCC_MR_ algorithm obtained the EDK_ms_(${\overline{\rho}}_{\mathbf{s},\mathbf{r}}$) by linear interpolation between EDK_ms_(${\rho}_i$) and EDK_ms_(${\rho}_j$) (${\rho}_i<{\overline{\rho}}_{\mathbf{s},\mathbf{r}}<{\rho}_j,$*i* or *j* = 0.25, 0.5, …, 2.0 g·cm^−3^). For instance, if the average mass density from **s** to **r** was 0.33 g·cm^−3^, the CCC_MR_ algorithm referred to the EDK_ms_(0.33) interpolated from the EDKs generated in the water sphere that had a density of 0.25 and 0.5 g·cm^−3^, and the energy deposition was obtained from the point *p*_**s, r**_ in the interpolated EDK_ms_(0.33).

**Fig. 2:**
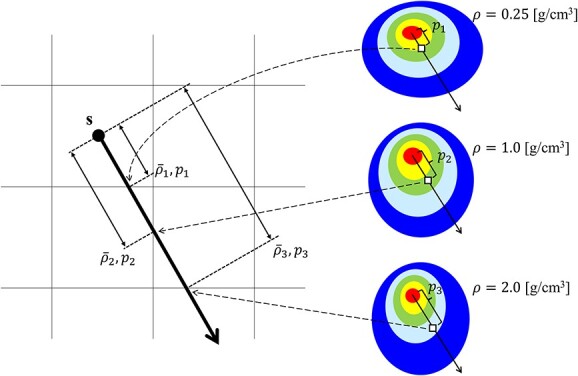
The schematic of the inhomogeneity correction in the CCC_MR_ algorithm. The left image shows the volume segment, and the right image shows the EDK_ms_ generated in each arbitrary mass density. **s** is the interaction point, and the thick arrow from **s** indicates the energy transportation direction. ${\overline{\rho}}_i$ and ${p}_i$ (*i* = 1, 2, 3) indicate the average mass density and the radiological path length from **s** to each cross plane, respectively. The image shows cases where ${\overline{\rho}}_1$, ${\overline{\rho}}_2$ and ${\overline{\rho}}_3$ are equal to 0.25, 1.0 and 2.0 g·cm^−3^, respectively. The energy depositions from **s** to ${p}_1$, ${p}_2$ and ${p}_3$ are obtained from ${p}_1$, ${p}_2$ and ${p}_3$ in the EDKs_ms_ generated at 0.25, 1.0 and 2.0 g·cm^−3^, respectively. When the EDK_ms_ is not preobtained for the mass density corresponding to ${\overline{\rho}}_i$, the EDK_ms_ corresponding to that mass density is calculated by linear interpolation and is used to obtain the energy deposition. In inhomogeneity correction in the CCC_MR_ algorithm, the referenced EDK_ms_ is determined or interpolated according to the average mass density from the interaction point to the calculation point.

Furthermore, the CCC_MR_ algorithm can be rewritten from Equation (7) as follows:


(9) \begin{equation*} D\left(\mathbf{r}\right)=\int \int \int \int{T}_E\left(\mathbf{s}\right){\mathrm{EDK}}_{\mathrm{ms}}\left(E,{\overline{\rho}}_{\mathbf{s},\mathbf{r}},{p}_{\mathbf{s},\mathbf{r}}\right){\mathrm{d}}^3s\mathrm{d}E \end{equation*}

where ${\mathrm{EDK}}_{\mathrm{ms}}\left(E,{\overline{\rho}}_{\mathbf{s},\mathbf{r}},{p}_{\mathbf{s},\mathbf{r}}\right)$ is the EDK_ms_ at the *p*_**s, r**_ of primary photon energy *E* obtained in the water sphere of mass density ${\overline{\rho}}_{\mathbf{s},\mathbf{r}}$. The schematic of the inhomogeneity correction in the CCC_MR_ algorithm is shown in Fig. 2. Our developed CCC_MR_ algorithm was described by FORTRAN and used Open-MPI to achieve parallel computation on a Central Processing Unit.

### Validation of the accuracy of inhomogeneity correction by density scaling

The CCC_MR_ algorithm proposed in this study uses density scaling and reference to EDK_ms_ according to the average mass density between interaction and dose calculation points as the inhomogeneity correction. The accuracy of the inhomogeneity correction was validated by comparing the dose distributions and profiles of spherical phantom obtained from the MC and CCC_MR_. The phantom was filled with water, and a 1-cm thick low mass density (0.25 [g/cm^3^]) slab was inserted at a depth of 0.5–1.5 cm. The dose distributions were obtained by interacting the primary photon 4 MeV at the center of sphere.

### Validation of the CCC_MR_ algorithm by comparison with the MC simulation

**Fig. 3:**
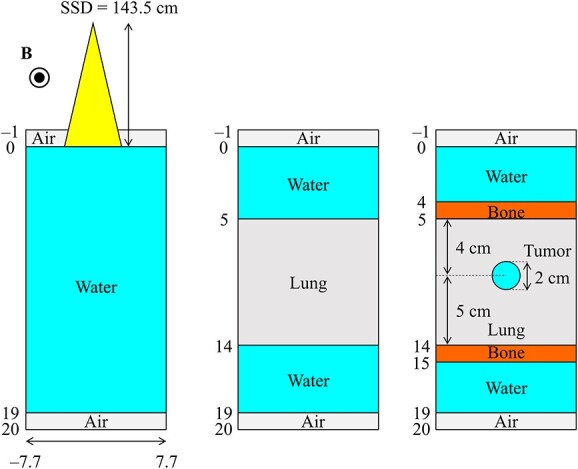
Schematic representation of phantom geometries. The left image shows the homogeneous water phantom, the center image shows the slab lung and water phantom and the right image shows the slab lung and bone phantom with an inserted spherical tumor.

In this study, three different virtual phantoms were used, each having dimensions of 21.0 × 15.4 × 15.4 cm^3^ with a voxel size of 0.2 cm. The schematic of the phantoms is shown in Fig. 3. One phantom was water-filled and homogeneous, and two were heterogeneous and comprised lung and bone slabs and a spherical tumor having a diameter of 2 cm. The mass densities of the water and tumor, the lung and the bone were 1, 0.33 and 1.92 g·cm^−3^, respectively.

Reference dose distributions were obtained from EGS5. As our proposed CCC_MR_ algorithm calculates dose to water (*D_w_*) by using the EDK_ms_ generated in water, EGS5 also calculates *D_w_*. *D_w_* was obtained using water as the medium in EGS5 and setting only the mass density as described above. The settings of the MC simulation, such as cutoff energy, EFRACH/EFRACL and field direction and strength, were similar for EDK_ms_ generation.

The source-to-surface distance and field sizes were set to 143.5 cm and 2 × 2, 5 × 5 and 10 × 10 cm^2^, respectively, at the phantom surface. The history number was set to 1 × 10^9^ for 2 × 2 and 5 × 5 cm^2^ and 2 × 10^9^ for 10 × 10 cm^2^. Variance reduction techniques to reduce the statistical uncertainty of the MC simulation were not used in our study. A point source was used to generate a photon beam composed of the Unity energy spectrum for MC calculations [6]. For the CCC_MR_ calculations, the energy spectrum was determined to produce the same percentage depth dose (PDD) curve as the PDD curve in the water phantom obtained from the MC calculation, and all CCC_MR_ calculations were performed using this energy spectrum with a point source. Neither the MC nor CCC_MR_ calculations considered varying the energy spectrum in the off-axis direction. All dose distributions obtained from both algorithms were normalized by the maximum dose point on the central axis (CAX) for each field size in the water phantom.

Volumetric dose comparison was performed using a 3D gamma test [21]. Furthermore, MATLAB software (MathWorks, Natick, MA), which was created by Mark Geurts (https://github.com/mwgeurts/gamma), was used to perform gamma analysis. The gamma index for each voxel at **r** is defined as follows:


$$\mathrm{\gamma} \left({\mathbf{r}}_{\mathrm{MC}}\right)=\min \left\{\Gamma \left({\mathbf{r}}_{{\mathrm{CCC}}_{\mathrm{MR}}},{\mathbf{r}}_{\mathrm{MC}}\right)\right\}\forall \left\{{\mathbf{r}}_{{\mathrm{CCC}}_{\mathrm{MR}}}\right\}$$

(10) \begin{equation*} \Gamma \left({\mathbf{r}}_{{\mathrm{CCC}}_{\mathrm{MR}}},{\mathbf{r}}_{\mathrm{MC}}\right)=\sqrt{\frac{{\left|{\mathbf{r}}_{{\mathrm{CCC}}_{\mathrm{MR}}}-{\mathbf{r}}_{\mathrm{MC}}\right|}^2}{\Delta{d}^2}+\frac{{\left[D\left({\mathbf{r}}_{{\mathrm{CCC}}_{\mathrm{MR}}}\right)-D\left({\mathbf{r}}_{\mathrm{MC}}\right)\right]}^2}{\Delta{D}^2}} \end{equation*}

where ${\mathbf{r}}_{\mathrm{MC}}$ and ${\mathbf{r}}_{{\mathrm{CCC}}_{\mathrm{MR}}}$ represent the position vectors for the reference (MC simulation) and evaluated (CCC_MR_ algorithm) distributions, respectively, and *D* represents the dose at each point. ∆*d* and ∆*D* are the distance to agreement (DTA) and dose difference criteria, respectively. Gamma values less than or equal to 1 and those greater than 1 indicate that the comparison passed and failed, respectively. The baseline criteria for ∆*d* and ∆*D* were 2 mm and 3%, respectively. As our comparisons were all between simulated doses, a tolerance of DTA 2 mm, which corresponds to one voxel size, was set. Gamma tests were performed for not only the entire volume, but also for each subregion of the dose distribution, which was divided into the buildup region, penumbra region and inner region, as in AAPM TG-53 definitions [22]. The buildup region corresponded to the voxels located from the surface to the depth of maximum dose (*d*_max_). The penumbra region corresponded to the voxels with doses between 20 and 80% of the CAX MC dose. The inner region corresponded to the voxels with doses greater than 80% of the CAX MC dose. The agreement between both algorithms was evaluated by the gamma passing rate, which was calculated by the ratio of the number of passing voxel to the total volume of each region.

All MC and CCC_MR_ simulations were performed in parallel on 40 cores. MC simulations were performed with different random number seeds on each core. A PC with two Intel Xeon Silver 4116 processors (2.1 GHz, 12 cores) was adopted for dose calculation, using the CCC_MR_ algorithm and MC simulation, and the calculation time was compared.

## RESULTS

**Fig. 4:**
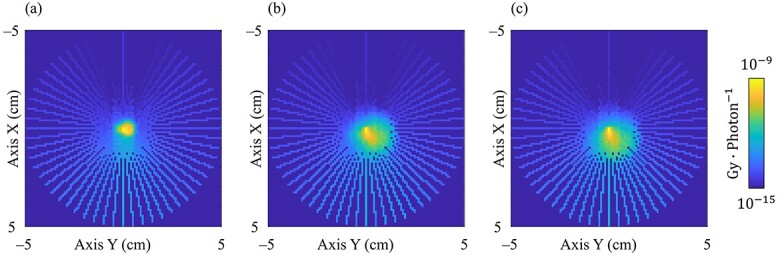
Generated 3D EDK of the primary photon energy at 4 MeV in the XY plane at the origin as shown in Fig. 1. The primary photon interacts at the center of the image. (**a**–**c**) show the EDKs generated in 0.25, 1.0 and 2.0 g·cm^−3^ water, respectively. WW, 10^−15^–10^−9^ Gy photon^−1^.

Figure 4 shows the EDKs within 5 g·cm^−2^ from the origin at 4.0 MeV in 0.25, 1.0 and 2.0 g·cm^−3^ water in the XY plane as shown in Fig. 1. The asymmetric EDKs were reconstructed, and the shape of the kernels varied with the mass density of water.

**Fig. 5:**
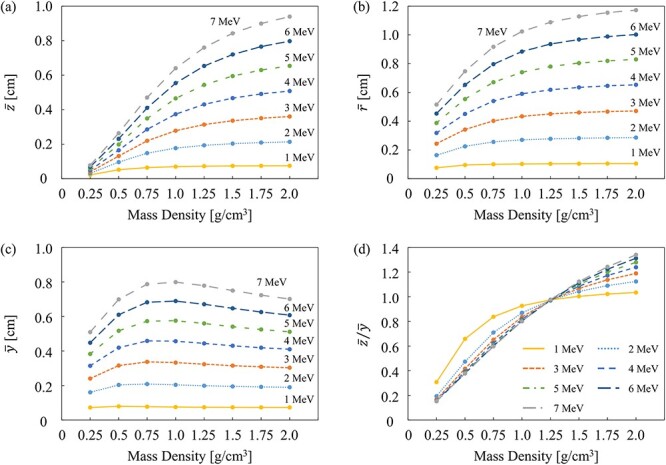
Comparison of the (**a**) effective depth, (**b**) effective radial distance, (**c**) effective lateral distance and (**d**) ratio of z/y in each mass density of water and at each primary photon energy. The statistical uncertainties of the MC simulation were smaller than the plot markers and were not visible on the graphs.

In Fig. 5, the parameters characterizing the EDK_ms_, the effective depth ($\overline{z}$), the effective radial distance ($\overline{r}$), the effective lateral distance ($\overline{y}$) and the ratio of $\overline{z}/\overline{y}$ are presented in each mass density of water at the primary photon energy from 1.0 to 7.0 MeV. The statistical uncertainties of the MC simulation were smaller than the plot markers and were not visible on the graphs. The effective depth and range increased as a function of the mass density. For the effective lateral distance, the curves produced a peak of ~0.75–1.0 g·cm^−3^. The ratio of $\overline{z}/\overline{y}$ indicated the direction in which charged particles deposited their energy in the medium. The EDK_ms_ was distorted to the left and right when the density was low and elongated vertically as the density increased.

**Fig. 6:**
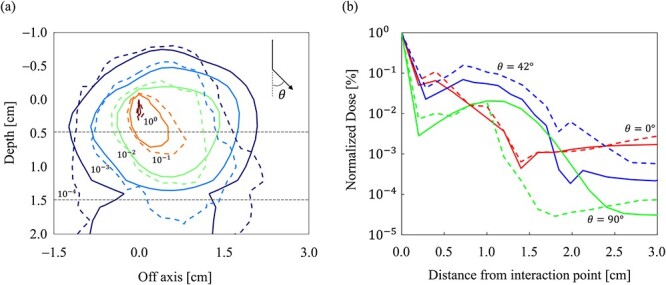
Incorporating a low mass density slab into the spherical water phantom allowed for a comparison of the inhomogeneity corrections of the MC and CCC_MR_. A comparison of the (**a**) dose distributions and (**b**) dose profiles, respectively. In the area enclosed by the dashed line in (a), a slab of low mass density is inserted. The MC and CCC_MR_ calculations are represented by solid and dashed lines, respectively. Dose distributions were normalized at the dose of primary photon interaction point (0, 0) in (a), and isodose lines are shown at 10^0^, 10^−1^, 10^−2^, 10^−3^ and 10^−4^. The dose profiles at 0°, 42° and 90° to the direction of the primary photon are depicted.

Figure 6 shows the dose distribution and profile obtained from the MC and CCC_MR_ in the spherical water phantom. In Fig. 6a, the isodose lines of the MC and CCC_MR_ are indicated by solid and dashed lines, respectively. Figure 6b shows the MC and CCC_MR_ dose profiles at 0°, 42° and 90° relative to the direction of the primary photon with solid and dashed lines, respectively. The dose profiles of the CCC_MR_ and MC were consistent in the 0° direction, but the CCC_MR_ overestimated and underestimated one of the MCs in the 42° and 90° directions, respectively.

**Fig. 7:**
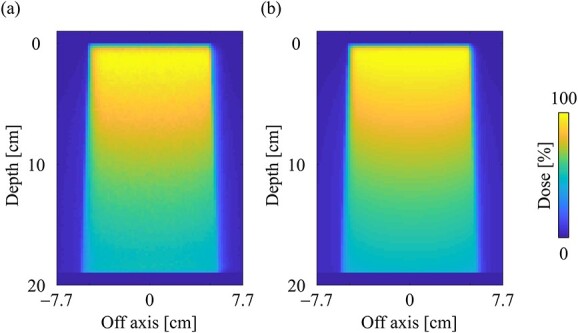
Dose distributions in the water phantom in the central slice obtained from the (**a**) MC and (**b**) CCC_MR_ techniques, respectively. Dose distributions were normalized by the maximum dose point on the CAX for each algorithm, and the same points were used for the normalization of other simulation phantoms. The field size was set to 10 × 10 cm^2^ at the phantom surface.

**Fig. 8:**
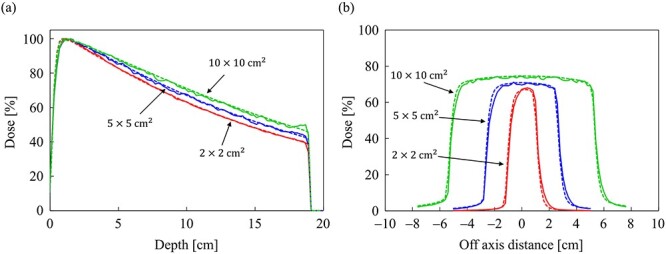
Comparison of (**a**) the PDD curves at the CAX and (**b**) off-axis profiles at a depth of 9 cm for the field sizes of 2 × 2, 5 × 5 and 10 × 10 cm^2^ in the water phantom. The solid and dash lines show MC and CCC_MR_ calculations, respectively.

Figure 7a and b shows the dose distributions obtained from the MC and CCC_MR_ with the field size of 10 × 10 cm^2^ in the water phantom, respectively. All dose distributions were normalized at the maximum dose point in the water phantom. Figure 8a and b shows the PDD curves at the CAX and the off-axis ratio (OAR) profiles at a depth of 9 cm obtained from both the MC and CCC_MR_ for each field size. Red, blue and green colors indicate the results for field sizes of 2 × 2, 5 × 5 and 10 × 10 cm^2^, respectively. Solid and dashed lines indicate the MC and CCC_MR_ results, respectively. All PDD curves obtained from the CCC_MR_ algorithm were in agreement with the MC curves, and the average differences from the MC simulation downstream of *d*_max_ were at the most 1.2%. The CCC_MR_ algorithm can simulate the left–right distorted asymmetry dose distributions that are characteristic of the transverse magnetic field, as shown in the OAR profiles. The average difference was between 1.8 and 4.3% in the inner of the full width at half maximum.

**Fig. 9:**
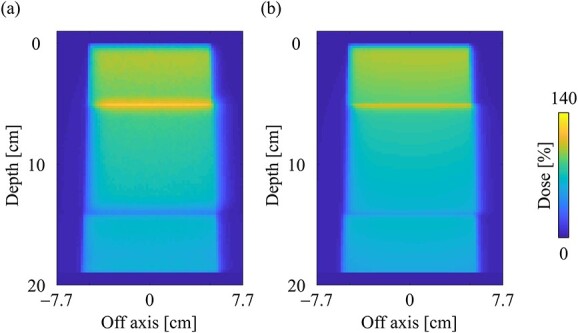
Dose distributions in the slab lung phantom in the central slice obtained from the (**a**) MC and (**b**) CCC_MR_ techniques, respectively. Dose distributions were normalized by the maximum dose point on the CAX in the water phantom. The field size was set to 10 × 10 cm^2^ at the phantom surface.

**Fig. 10:**
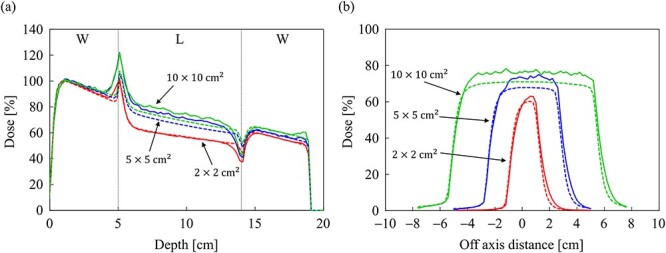
Comparison of (**a**) the PDD curves at the CAX and (**b**) off-axis profiles at a depth of 9 cm for the field sizes of 2 × 2, 5 × 5 and 10 × 10 cm^2^ in the slab lung phantom. The abbreviations W and L in (a) stand for water and lung, respectively. The solid and dash lines show MC and CCC_MR_ calculations, respectively.

Figure 9a and b shows the dose distributions obtained from the MC and CCC_MR_ with the field size of 10 × 10 cm^2^ in the slab lung phantom. The CCC_MR_ algorithm can simulate hot and cold spots caused by changing the electron trajectories at the mass density boundaries, similar to that with MC simulation. Figure 10a and b shows the PDD curves at the CAX and the OAR profiles at a depth of 9 cm in the lung obtained from both MC and CCC_MR_ for each field size. Red, blue and green colors indicate that the field sizes were 2 × 2, 5 × 5 and 10 × 10 cm^2^, respectively. The results of the MC and CCC_MR_ were represented by dashed lines and solid lines, respectively. Although the CCC_MR_ algorithm calculated the PDD curves consistent with the MC simulation in the water, the trend in the lungs differed depending on the field size. In the lung, the CCC_MR_ algorithm calculated the same dose as the MC simulation for the field size of 2 × 2 cm^2^, whereas it underestimated the dose by 10% for the field sizes of 5 × 5 and 10 × 10 cm^2^. The OAR profiles also showed underestimation within the irradiation field in the lung. The CCC_MR_ algorithm also underestimated the hot and cold spots occurring in the water/lung boundaries relative to the MC simulation.

**Fig. 11:**
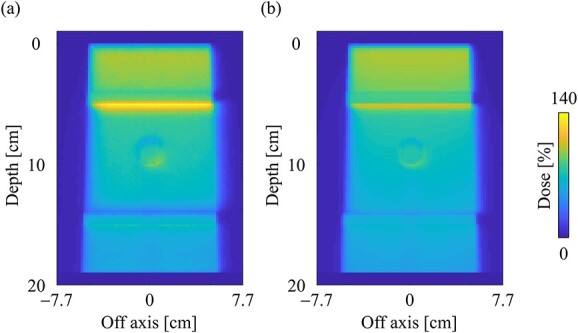
Dose distributions in the spherical tumor-inserted slab phantom in the central slice obtained from the (**a**) MC and (**b**) CCC_MR_ techniques, respectively. Dose distributions were normalized by the maximum dose point on the CAX in the water phantom. The field size was set to 10 × 10 cm^2^ at the phantom surface.

**Fig. 12:**
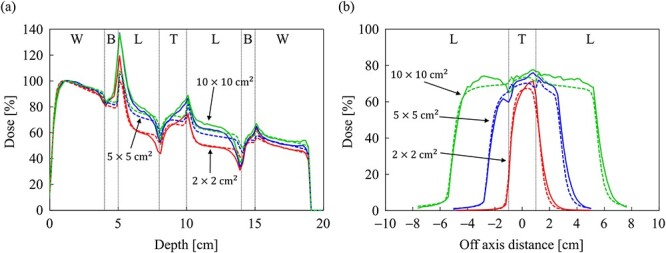
Comparison of (**a**) the PDD curves at the CAX and (**b**) off-axis profiles at a depth of 9 cm for the field sizes of 2 × 2, 5 × 5 and 10 × 10 cm^2^ in the spherical tumor-inserted slab phantom. The abbreviations W, B, L and T in (a) and (b) stand for water, bone, lung and tumor, respectively. The solid and dash lines show MC and CCC_MR_ calculations, respectively.

The dose distributions obtained from the MC and CCC_MR_ with the field size of 10 × 10 cm^2^ in a slab phantom inserted with a spherical tumor are depicted in Fig. 11a and b, respectively. The CCC_MR_ algorithm can simulate the dose changes at the mass density boundaries, similar to that with the MC simulation. The PDD curves at the CAX and the OAR profiles at a depth of 9 cm (the center of the tumor) obtained from the MC and CCC_MR_ at each field size are depicted in Fig. 12a and b, respectively. The results for a field size of 2 × 2, 5 × 5 and 10 × 10 cm^2^ were represented by the colors red, blue and green, respectively. Moreover, the results of the MC and CCC_MR_ were represented by dashed lines and solid lines, respectively. The abbreviations W, B, L and T in Fig. 12 represent water, bone, lung and tumor, respectively. The PDD curves showed similar results to those of the slab lung phantom in the lung region of the spherical tumor phantom, with the CCC_MR_ and MC doses agreeing in the field size of 2 × 2 cm^2^, but showing an underestimation of close to 10% with the other field sizes. In bone and tumor regions, the CCC_MR_ algorithm underestimated the dose relative to the MC simulation regardless of the field size.

**Fig. 13:**
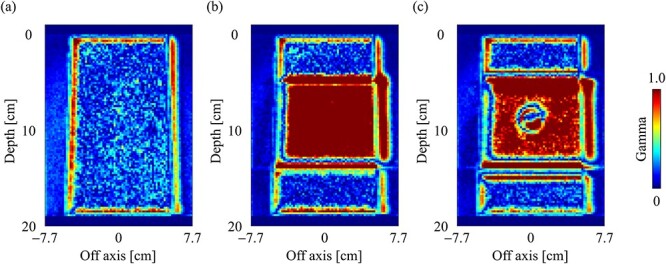
Gamma distribution (3%/2 mm) of dose distributions with the field size of 10 × 10 cm^2^ in the central slice for (**a**) water, (**b**) slab lung and (**c**) spherical tumor-inserted slab phantom.

The gamma distributions of the central plane for the water, slab lung and spherical tumor-inserted slab phantom are depicted in Fig. 13a–c, respectively, with a field size of 10 × 10 cm^2^. There was a notable number of voxels in the bone and lung regions in the inhomogeneity phantoms, and the findings did not pass the 3%/2 mm gamma criterion. Table 1 shows the gamma passing rate for each phantom, field size and subregion. In the water phantom, the gamma passing rates were >99%, regardless of the field size, in all regions, except for the buildup region. In the inhomogeneity phantoms, the gamma passing rates were > 90% in the buildup and penumbra regions, but 58.92–97.64% in the inner regions. Differences occurred mainly in the bone and lung regions, as shown by the dose profiles and gamma distributions.

**Table 1:** The percentage passing rate of 3D gamma indices in the three phantoms for each subregion and field size; the criteria of dose difference and DTA were set to 3% and 2 mm, respectively

		Field size (cm^2^)		
Phantom	Subregion	2 × 2	5 × 5	10 × 10
Water	Buildup	100.0	93.8	97.6
	Penumbra	100.00	99.5	99.7
	Inner	100.00	99.6	99.9
	All	99.9	99.2	99.7
Slab lung	Buildup	98.7	98.2	98.7
	Penumbra	97.4	95.7	93.7
	Inner	97.6	69.8	58.9
	All	97.5	80.2	68.9
Spherical tumor	Buildup	98.9	97.8	98.1
	Penumbra	98.0	96.0	93.6
	Inner	93.9	80.8	76.0
	All	96.1	86.7	81.2

The comparison of the computation time between the MC simulation and CCC_MR_ algorithm is shown in Table 2. Speed increases due to the use of the CCC_MR_ algorithm were ~150–1900 times depending on the field size.

**Table 2:** Comparison of the computation times between the MC and CCC_MR_ techniques in the three phantoms; the unit of time is minutes

Phantom	Field size (cm^2^)	MC	CCC_MR_	Speed increase
Water	2 × 2	1118.6	1.2	×908
	5 × 5	1101.5	6.7	×165
	10 × 10	10 793.4	33.1	×326
Slab lung	2 × 2	1099.4	1.1	×980
	5 × 5	1106.8	6.1	×182
	10 × 10	10 779.1	30.6	×353
Spherical tumor	2 × 2	2225.8	1.2	×1906
	5 × 5	2226.8	6.3	×354
	10 × 10	10 786.9	31.4	×344

## DISCUSSION

Generally, in the CCC algorithm without a magnetic field, the algorithm uses an EDK for each energy, which is generated in water with a mass density of 1 g·cm^−3^ and considers inhomogeneity correction by applying density scaling for each ray. In a transverse magnetic field, the trajectory of the charged particles varies due to the change in the mass density, as described above, distorting EDKs and dose distributions. To reproduce this phenomenon in the CCC algorithm, first, we generated the distorted EDKs in water with various mass densities (EDK_ms_) in a transverse magnetic field. Second, the energy deposition to each point was determined by referring to the EDKs_ms_ according to the average mass density from the interaction point to the calculation point. In addition, if necessary, the EDK_ms_ corresponding to the average mass density was obtained by interpolation and applied to the dose calculations.

The CCC_MR_ algorithm proposed in this study can calculate the dose distribution with left–right distortion that is characteristic of the transverse magnetic fields by applying EDKs_ms_. Furthermore, although the CCC_MR_ algorithm was able to reproduce the dose change caused by the abrupt change in electron trajectories at the mass density boundaries, the dose change was underestimated relative to the MC simulation. The gamma passing rates (3%/2 mm) in the water phantom were greater than 99% for all field sizes in all regions, except the buildup region, indicating that the CCC_MR_ algorithm can calculate dose distributions consistent with the MC simulation in homogeneity regions. The CCC_MR_ algorithm underestimated the dose by 10% in the low mass density region for larger field sizes, markedly reducing the gamma passing rate. However, the CCC_MR_ algorithm calculated the doses consistent with the MC simulation even in the low mass density region for the field size of 2 × 2 cm^2^, and the gamma passing rates were over 97% for the slab lung and 93% for the slab phantom with a spherical tumor in the central region. The gamma passing rates of the entire region with the field size of 2 × 2 cm^2^ were 97 and 96% or higher for the two heterogeneous phantoms, respectively, indicating the possibility of the clinical application of the CCC_MR_ algorithm in dose calculation for small field sizes. In the MC, the energy spectrum was defined between 0.07 and 7.0 MeV at 0.07 MeV intervals. In contrast, in the CCC_MR_, the energy spectrum was defined at 1 MeV intervals between 1.0 and 7.0 MeV in order to make the PDD curve after buildup coincide with the MC. The difference in the definition of the energy spectrum between MC and CCC_MR_ appeared as a change in the rise of the buildup region of the PDD curve. The EDKs_ms_ generated by averaging the energy depositions over a 3D con-shaped space and have an angular resolution of 6°. The degree of distortion is also averaged within this angular range. The difference in the penumbra shape between the MC and CCC_MR_ is assumed to be due to distortion averaging in generating the EDKs_ms_.

In a spherical water phantom with a low mass density slab region, the dose distributions of the MC and CCC_MR_ were compared to determine the accuracy of the CCC_MR_ inhomogeneity correction. In terms of the primary photon direction, the dose profiles of the MC and CCC_MR_ were in agreement with each other in the 0° direction, whereas the CCC_MR_ overestimated and underestimated the direction in the 42° and 90° directions, respectively. The trajectory of a charged particle in a magnetic field varies with the mass density of the medium. According to Bouchard *et al*., in a uniform magnetic field, the radius of the circular orbit of a charged particle decreases as the mass density of the medium decreases [23]. However, the proposed CCC_MR_ assumes that energy is transferred radially and linearly from the interaction point and does not consider changes in the trajectory caused by changes in mass density. This may have caused dose difference in low mass density regions and at the boundary of mass density changes. Furthermore, a previous study found that density scaling may not be sufficient to eliminate inhomogeneities in larger field sizes [14]. Similarly, the present study found that as the field size increased, the gamma passing rate decreased in low-density regions.

To characterize the EDK_ms_ in each mass density at each energy, the effective depth ($\overline{z}$), radial distance ($\overline{r}$), lateral distance ($\overline{y}$) and $\overline{z}/\overline{y}$ ratio were evaluated. The effective depth and radial distance increased with the water mass density. The increase in the density of the medium caused the charged particles to be less sensitive to the Lorentz force; hence, the charged particles were transported radially in the depth or radial direction without a curve. As a result, the shape of the kernel ($\overline{z}/\overline{y}$) varied with the water mass density.

AAPM Task Group 85 reported that the total uncertainty in dose prescription to a patient, including many considerations, should be <5% and that it is realistic to achieve 3% in the future from the time of publication, in which case the accuracy of dose calculation should be ~1–2% [24]. The results of this study indicate that the CCC_MR_ algorithm can calculate the dose distribution in water with a difference of <2% compared with the MC simulation, except in the mass density change regions. The CCC_MR_ algorithm may be used for independent verification of MRgRT in regions, such as the head and pelvis, which are low-density areas with densities close to that of water, after understanding the characteristic of the underestimation of the dose in low-density regions. However, comparisons under more realistic conditions using patient CT images are mandatory.

Some researchers developed and validated the GPU-based MC dose engine (GPU-MC) for MRgRT. Li *et al*. validated the GPU-MC dose engine (gDPM) and revealed that the gDPU requires <40 s with a statistical uncertainty of 1% to obtain the dose distribution [25]. Hissoiny *et al*. reported that the GPU-based MC dose calculation platform calculates the dose in a patient with prostate disease in <56 s with a statistical uncertainty of 1% for one beam when using two GPUs [26]. Our proposed CCC_MR_ algorithm calculates the dose distribution with parallel computation on the CPU and needs more time to obtain the dose distribution when compared with the GPU-MC. However, as reported by other researchers, our proposed CCC_MR_ algorithm can be extended to GPU-based parallel computing; hence, adapting the CCC_MR_ algorithm to the GPU-based algorithm would solve the computation time problem in the future [11, 12, 27, 28]. Assuming that the computation time problem is solved, the proposed CCC_MR_ algorithm could be used not only as an independent verification system, but also for the optimization of IMRT during online-ART and real-time dosimetry during patient treatment.

Pfaffenberger *et al*. reported a method for obtaining the dose distributions in a magnetic field by applying kernel convolutions [29]. They proposed a method using a point kernel that analytically reproduces the trajectory of the Compton electron in a magnetic field and a warping method that transforms the dose distributions without a magnetic field into the distributions in a magnetic field. The former method was found to be difficult to apply to dose calculation because it was not able to take into account the effect of scatter doses generated by the Compton electrons. Moreover, they concluded that the latter method could reproduce the left–right distorted dose distributions due to the magnetic field, but it was difficult to model the dose variation due to ERE, and the approach was suitable for dose calculations in a homogeneous medium, such as the brain, or within optimization calculations. The CCC_MR_ algorithm proposed in this study was a modified version of the conventional CCC method used in clinical practice for dose calculation. The conventional CCC method uses a 2D EDK, but the CCC_MR_ algorithm uses a 3D EDK to reproduce the distorted distribution in the magnetic field. In addition, the density scaling method has been used to correct for inhomogeneity in CCC methods, but in the CCC_MR_ algorithm, the referenced EDK was also changed according to the average mass density from the interaction point to the calculation point [14]. The modifications enabled not only the calculation of the left–right distorted dose distributions characteristic of a transverse magnetic field, but also hot/cold spots caused by changes in the electron trajectory to be reproduced at mass density boundaries.

This study has several limitations. First, the voxel size was fixed at 2 mm, and the variation in accuracy by voxel size was not verified. Second, only a differential kernel (DK) was used as the EDK. Lu *et al*. reported that a DK can calculate the dose distributions in a short time. Conversely, the accuracy deteriorates as the voxel size increases [19]. Moreover, a cumulative−cumulative kernel can obtain stable results regardless of the voxel size, although it increases the computation time. Finally, the angular resolution of the EDK_ms_ used in this study was set to 6° only, and the calculation accuracy when a higher resolution EDK_ms_ was used was not evaluated. The maximum angular resolution was 6° owing to the limited memory capacity of the PC used for the dose calculation. Thus, the dose calculation was performed with this setting. However, it is obvious that the kernel resolution affects the CCC dose calculation accuracy, and the effect of angular resolution on dose distribution should be revealed. The dependency of the voxel size and the effect of the types and angular resolutions of EDKs on dose distributions are subjects of future studies.

## CONCLUSION

This study proposed the CCC_MR_ algorithm that allows calculation of left–right distorted asymmetric dose distributions for MRgRT. The CCC_MR_ algorithm can correct inhomogeneity in the simulation geometry by using 3D distorted EDKs in a transverse magnetic field obtained with the arbitrary mass density of water. Moreover, the CCC_MR_ algorithm obtains the energy deposition by referring to the EDKs_ms_ obtained by interpolation according to the average mass density from the interaction point to the calculation point. Compared with the results of MC simulation, the CCC_MR_ algorithm can obtain dose distributions indicating a gamma passing rate over 96% with small field sizes, although there is 10% underestimation with relatively larger field sizes in low mass density regions. The current CCC_MR_ algorithm uses CPU-based parallel computation and requires a longer time for obtaining the dose distributions when compared with GPU-MC. However, as the CCC_MR_ algorithm can also be adapted for parallel computation using GPUs, the computation time problem would be solved in the future. The CCC_MR_ algorithm is currently under development and would be applied for not only independent verification, but also for real-time dosimetry of MRgRT in the future.

## Data Availability

The data underlying this article will be shared on reasonable request to the corresponding author.

## References

[1] Dieterich S, Green O, Booth J. SBRT targets that move with respiration. Phys Med 2018;56:19–24. 30527085 10.1016/j.ejmp.2018.10.021

[2] Yang Y, Cao M, Sheng K et al. Longitudinal diffusion MRI for treatment response assessment: preliminary experience using an MRI-guided tri-cobalt 60 radiotherapy system. Med Phys 2016;43:1369–73. 26936721 10.1118/1.4942381 PMC6961701

[3] Datta A, Aznar MC, Dubec M et al. Delivering functional imaging on the MRI-Linac: current challenges and potential solutions. Clin Oncol (R Coll Radiol) 2018;30:702–10. 30224203 10.1016/j.clon.2018.08.005

[4] Acharya S, Fischer-Valuck BW, Kashani R et al. Online magnetic resonance image guided adaptive radiation therapy: first clinical applications. Int J Radiat Oncol Biol Phys 2016;94:394–403. 26678659 10.1016/j.ijrobp.2015.10.015

[5] Winkel D, Bol GH, Kroon PS et al. Adaptive radiotherapy: the Elekta Unity MR-linac concept. Clin Transl Radiat Oncol 2019;18:54–9. 31341976 10.1016/j.ctro.2019.04.001 PMC6630157

[6] Ahmad SB, Sarfehnia A, Paudel MR et al. Evaluation of a commercial MRI Linac based Monte Carlo dose calculation algorithm with GEANT4. Med Phys 2016;43:894–907. 26843250 10.1118/1.4939808

[7] Mutic S, Dempsey JF. The ViewRay system: magnetic resonance-guided and controlled radiotherapy. Semin Radiat Oncol 2014;24:196–9. 24931092 10.1016/j.semradonc.2014.02.008

[8] Hissoiny S, Ozell B, Bouchard H et al. GPUMCD: a new GPU-oriented Monte Carlo dose calculation platform. Med Phys 2011;38:754–64. 21452713 10.1118/1.3539725

[9] Raaymakers BW, Jurgenliemk-Schulz IM, Bol GH et al. First patients treated with a 1.5 T MRI-Linac: clinical proof of concept of a high-precision, high-field MRI guided radiotherapy treatment. Phys Med Biol 2017;62:L41–50. 29135471 10.1088/1361-6560/aa9517

[10] Muinck Keizer DM, Willigenburg T, der Voort van Zyp JRN et al. Seminal vesicle intrafraction motion during the delivery of radiotherapy sessions on a 1.5 T MR-Linac. Radiother Oncol 2021;162:162–9. 34293410 10.1016/j.radonc.2021.07.014

[11] Jacques R, Wong J, Taylor R et al. Real-time dose computation: GPU-accelerated source modeling and superposition/convolution. Med Phys 2011;38:294–305. 21361198 10.1118/1.3483785

[12] Neylon J, Sheng K, Yu V et al. A nonvoxel-based dose convolution/superposition algorithm optimized for scalable GPU architectures. Med Phys 2014;41:101711. 25281950 10.1118/1.4895822

[13] Chen X, Prior P, Chen GP et al. Technical note: dose effects of 1.5 T transverse magnetic field on tissue interfaces in MRI-guided radiotherapy. Med Phys 2016;43:4797. 27487897 10.1118/1.4959534

[14] Woo MK, Cunningham JR. The validity of the density scaling method in primary electron transport for photon and electron beams. Med Phys 1990;17:187–94. 2333045 10.1118/1.596497

[15] Heidarloo N, Aghamiri SMR, Saghamanesh S et al. Generation of material-specific energy deposition kernels for kilovoltage x-ray dose calculations. Med Phys 2021;48:5423–39. 34173989 10.1002/mp.15061

[16] Huang JY, Eklund D, Childress NL et al. Investigation of various energy deposition kernel refinements for the convolutionsuperposition method. Med Phys 2013;40:121721. 24320507 10.1118/1.4831758 PMC3856653

[17] Ito K, Kadoya N, Katsuta Y et al. Evaluation of the electron transport algorithm in magnetic field in EGS5 Monte Carlo code. Phys Med 2022;93:46–51. 34922223 10.1016/j.ejmp.2021.12.001

[18] Mackie TR, Bielajew AF, Rogers DW et al. Generate EDK. Phys Med Biol 1988;33:1–20. 10.1088/0031-9155/33/1/001 3353444

[19] Lu W, Olivera GH, Chen ML et al. Accurate convolution/superposition for multi-resolution dose calculation using cumulative tabulated kernels. Phys Med Biol 2005;50:655–80. 15773626 10.1088/0031-9155/50/4/007

[20] Reckwerdt PJ, Mackie TR. Superposition/convolution speed improvements using run-length raytracing. Med Phys 1992;19:784–84.

[21] Low DA . Gamma dose distribution evaluation tool. J Phys Conf Ser 2010;250:012071. 10.1088/1742-6596/250/1/012035 PMC3015239 21218172

[22] Fraass B, Doppke K, Hunt M et al. American Association of Physicists in Medicine radiation therapy committee task group 53: quality assurance for clinical radiotherapy treatment planning. Med Phys 1998;25:1773–829. 9800687 10.1118/1.598373

[23] Bouchard H, de Pooter J, Bielajew A et al. Reference dosimetry in the presence of magnetic fields: conditions to validate Monte Carlo simulations. Phys Med Biol 2015;60:6639–54. 26271015 10.1088/0031-9155/60/17/6639

[24] Papanikolaou N, Battista JJ, Boyer AL et al. Tissue inhomogeneity corrections for megavoltage photon beams. AAPM Task Group #65 Radiation Therapy Committee. 2004.

[25] Li Y, Ding S, Wang B et al. Extension and validation of a GPU-Monte Carlo dose engine gDPM for 1.5 T MR-LINAC online independent dose verification. Med Phys 2021;48:6174–83. 10.1002/mp.15165 34387872

[26] Hissoiny S, Raaijmakers AJ, Ozell B et al. Fast dose calculation in magnetic fields with GPUMCD. Phys Med Biol 2011;56:5119–29. 21775790 10.1088/0031-9155/56/16/003

[27] Jacques R, Taylor R, Wong J et al. Towards real-time radiation therapy: GPU accelerated superposition/convolution. Comput Methods Prog Biomed 2010;98:285–92. 10.1016/j.cmpb.2009.07.004 19695731

[28] Chen Q, Chen M, Lu W. Ultrafast convolution/superposition using tabulated and exponential kernels on GPU. Med Phys 2011;38:1150–61. 21520827 10.1118/1.3551996

[29] Pfaffenberger A, Oelfke U. Dose Calculation Algorithms for Radiation Therapy with an MRI-Integrated Radiation Device. Universitätsbibliothek Heidelberg, 2013.

